# Some American Hospitals

**Published:** 1892-01-16

**Authors:** 


### SOME AMERICAN HOSPITALS.

By Our Special Commissioner.

### III.—SALT LAKE CITY.

Salt Lake City, the home of the Mormon Saints is pleasantly situated, and, by an excellent system of irrigation, the Mormons have converted the desert into a fruitful garden, producing fruit and all kinds of trees in abundance. The result is that the city—which has increased enormously owing to a recent boom—presents a most attractive picture to the eye, as the streets are carefully laid out, the roadways excellently made, and the houses are for the most part unusually commodious, whilst many of them are placed in the centre of a six acre piece. The sign of the Mormons is a bee-hive, signifying industry, and Brigham Young's houses may be easily recognised from the fact that each house has a bee-hive at the top. It was the policy of the early founders of the city to make every inhabitant industrious, and with this object, the frontage of each block was divided into three parts, so that a house might be placed at each corner, and one in the middle, which gave an abundant acreage to each of them. The Prophet's instructions were to cultivate fruit trees, and, in many of the streets and avenues numerous apple, pear, peach, and plum trees are seen, all flourishing and in full bearing. In Salt Lake City, as elsewhere throughout America, the majority of the streets and avenues are traversed by the electric cars, which travel at the rate of about ten miles an hour, and enable a visitor to move about with rapidity and ease. Thus at San Francisco, owing to the hilly character of the city, it would be practically impossible to get about at all were it not for the electric cars, which run up and down hill at a rapid pace without let or hindrance.

About twenty miles from Salt Lake City is situated the great Salt Lake, the water of which contains 20 per cent. of salt, and nothing can live in it. At Garfield Beach the Union Pacific Railway Company have erected a hotel and bathing establishment, which is much frequented for five months of the year. The lake is unusually shallow, but, owing to the character of the water, anyone can float with ease, and owing to this circumstance swimming is by no means an easy task. The amount of salt in the water makes it very unpleasant, if not dangerous, to swallow. Serious injury has sometimes been done to the eyes from want of care on the part of the bathers. On Garfield Beach many trees have been planted, and here, at the present time, one of the few herds of buffalo to be seen in America is to be found. The animals are kept in a wild state, and the buffalo can be seen in his native element, charging about and galloping as he used to do formerly on the prairies.

Salt Lake City contains three hospitals—Holy Cross, St. Mark's, and Deseret. The two latter are small and unimportant, and we will, therefore, content ourselves by describing the first only. The Holy Cross Hospital is maintained by patients' payments and voluntary contributions. It is under the control of the municipal authorities, who make up any deficiency in the funds from year to year. It consists of upwards of 100 beds. The present building is new, having been recently erected, and is, on the whole, well adapted for the purpose to which it is put. It may be classed as a pavilion hospital of the old H type. Much excellent surgical work appears to be done here. The patients seem comfortable and well cared for. The nursing is undertaken by the Sisters of the Holy Cross—the headquarters of the Order being Nôtre Dame. Indiana. One of the Sisters, acts as dispenser. The whole aspect of the building shows that those responsible for the nursing and management take a pride in their work, to the no small benefit of the patients. There are many private wards for paying patients—those to the right being devoted to women, and those to the left to men, patients occupying single wards, the whole of which are well furnished and very comfortable, pay from 10 to 15 dollars per week, and, in addition, such fees for medical attendance as may be agreed upon. Many of the patients in the larger wards pay weekly sums according to their means.

The whole of the Sisters who act as nurses, and who also undertake the domestic management, sleep together in one large dormitory at the top of the hospital. Each Sister has a bed to herself, which is surrounded by white curtains on the old French plan. These Sisters appear to have very few comforts, and their sleeping accommodation is inadequate and out of character, having regard to the comfort in many other portions of the establishment. Sister Holy Cross acts as Directress, while Sister Bartholomew has charge of the household arrangements. A feature of this hospital is the carpets, which are put down in all the corridors as well as on the staircases. These carpets are taken up each day and beaten, and they are also disinfected and cleaned at regular intervals. We were informed that carpets were used in preference to linoleum because experience proved, that as most of the patients were miners, the heavy boots they were accustomed to wear destroyed the latter material, besides causing a great deal of noise when the convalescent patients walked about. The hospital has its own laundry, and has also an orphanage—situated in the hospital grounds—under the charge of the Sisters. Altogether, we should say that the administration of this hospital is far above that of the average of similar institutions in America, and that the people of Salt Lake City are fortunate in having the co-operation of the Sisters of the Holy Cross.

We may mention, in passing, that having regard to the account of the Salt Lake Lunacy Asylum, which appears in "Lunacy in Many Lands," issued by the Government of New South Wales, we thought it our duty to make enquiries about this establishment. So far as we could learn, no such establishment exists at the present time, at any rate, the nearest asylum being some twenty miles off. We were, therefore, unable to personally inspect an institution which, from the description in "Lunacy in Many Lands," may be justly characterised as the very worst establishment for the insane in the civilised world.

